# Unraveling Enhanced Superconductivity in Single-Layer FeSe through Substrate Surface Terminations

**DOI:** 10.1021/acs.nanolett.5c01298

**Published:** 2025-09-03

**Authors:** Qiang Zou, Gi-Yeop Kim, Jong-Hoon Kang, Basu Dev Oli, Zhuozhi Ge, Michael Weinert, Subhasish Mandal, Chang-Beom Eom, Si-Young Choi, Lian Li

**Affiliations:** † Department of Physics and Astronomy, 5631 West Virginia University, Morgantown, West Virginia 26506, United States; ‡ Department of Materials Science and Engineering, 540180 Pohang University of Science and Technology, Pohang 37673, Republic of Korea; § Department of Materials Science and Engineering, 5228 University of Wisconsin−Madison, Madison, Wisconsin 53706, United States; ∥ Department of Physics, University of Wisconsin, Milwaukee, Wisconsin 53211, United States; ⊥ Center for van der Waals Quantum Solids Institute for Basic Science, Pohang 37673, Republic of Korea

**Keywords:** FeSe, TiO_2_-terminated SrTiO_3_, SrO-terminated SrTiO_3_, scanning tunneling microscopy, electron correlations, Se−Fe−Se tetrahedral angle

## Abstract

Single-layer FeSe on SrTiO_3_(001) substrates shows a superconducting transition temperature much higher than that of bulk FeSe, which has been attributed to factors such as electron doping, interfacial electron–phonon coupling, and electron correlations. To pinpoint the primary driver, we grew single-layer FeSe films on SrTiO_3_(001) substrates with coexisting TiO_2_ and SrO surface terminations. Scanning tunneling spectroscopy revealed a larger superconducting gap (17.0 meV) on the TiO_2_-termination than on the SrO-termination (10.5 meV). Tunneling spectroscopy also showed a larger work function on the SrO surface, resulting in reduced charge transfer to FeSe, as confirmed by angle-resolved photoemission spectroscopy. Scanning transmission electron microscopy further revealed distinctive interfacial atomic-scale structures, with the Se–Fe–Se tetrahedral angle changing from 109.5° on the SrO-termination to 104.9° on the TiO_2_-termination. Compared to dynamical mean field theory calculations, our results indicate that enhanced superconductivity in single-layer FeSe/TiO_2_ arises from optimal electron correlations, in addition to sufficient charge transfer from the substrate.

## Graphical abstract

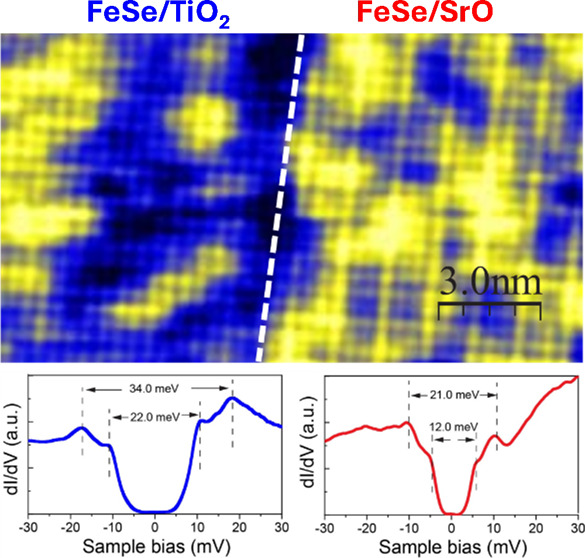

The sensitive dependence of two-dimensional (2D) materials on their environment often gives rise to emergent properties not seen in bulk forms. For films grown on a substrate, epitaxial constraints can generate 2D structural polymorphs that are otherwise prohibited in bulk crystals. For example, the metastable topological insulator 1T′-WSe_2_ can be grown on the SrTiO_3_(001) (STO) substrate using molecular beam epitaxy (MBE).^1^ Even more strikingly, single-layer (SL) FeSe epitaxially grown on the STO substrate has shown a superconducting transition temperature (*T*_C_) nearly 8-fold higher than that of bulk FeSe.^2–8^ Such enhancement can be further tailored by photoexcitation in the STO substrate after exposure to UV light.^9^ In addition to a 20% increase in the zero resistance *T*_C_, the UV light-induced superconducting state is also nonvolatile, which can also be rapidly reversed by applying voltage pulses to the STO substrate,^9^ demonstrating the optical and electrical control of quantum states at designed correlated interfaces of 2D materials and 3D complex oxides.

The mechanisms for the enhancement and control of *T*_C_ in the SL FeSe/STO are still a subject of intense debate, with proposed contributors including electron doping^3,6,10–16^ and strong electron–phonon coupling (EPC) involving the STO Fuchs–Kliewer phonons.^17–22^ While the evidence for the EPC, i.e., the appearance of replica bands in angle-resolved photoemission spectroscopy (ARPES) measurements,^17^ has been debated,^23^ recent work points to a significant role for localized phonons at the interfacial TiO_2_ layer.^24,25^ An electron doping of ∼0.10–0.12 e/Fe has been considered optimal for achieving high *T*_C_ at the FeSe/TiO_2_ interface.^15^ Furthermore, studies on the FeSe/FeO_*x*_ interface have shown that similar or even higher *T*_C_ can be achieved with only 40% of that doping level (0.038 e/Fe),^26,27^ suggesting that factors beyond electron doping can also contribute to the enhanced superconductivity. One such factor is electron correlations. Embedded dynamical mean-field theory (eDMFT) calculations have shown that these correlations in the SL FeSe/STO are orbital-dependent, with the d_*xy*_ orbital being the most correlated and controlled by the Se–Fe–Se tetrahedral angle and doping from interfacial oxygen vacancies.^28^

From an experimental perspective, a more intuitive way to isolate the effects of EPC, electron doping, and electron correlations in enhancing *T*_C_ is to grow epitaxial SL FeSe on the same substrate with different surface terminations. This approach eliminates the bulk phonon contributions and allows independent control of two other key parameters: 1) the surface work function, which affects charge transfer and doping, and 2) the atomic geometry of the FeSe tetrahedron, which controls electron correlations.

To this end, we grew SL FeSe on STO substrates with 100% TiO_2_ and mixed TiO_2_ and SrO surface terminations. Using scanning tunneling microscopy/spectroscopy (STM/S), we determined a surface work function of 3.8 and 4.3 eV on the TiO_2_ and SrO terminations and superconducting gap of Δ = 17.0 and 10.5 meV for FeSe/TiO_2_ and FeSe/SrO, respectively. Furthermore, scanning transmission electron microscopy (STEM) revealed distinct interfacial structures with an average Se–Fe–Se angle of 109.5° for FeSe/SrO and 104.9° for FeSe/TiO_2_. By comparing these experimental observations with eDMFT calculations, our findings demonstrate that the electron correlations are optimal at the FeSe/TiO_2_ interface for achieving a high *T*_C_.

As shown in Figure 1(a), both SrO and TiO_2_ terminations are allowed on the STO surface, separated by steps with a half-unit cell height in the *c*-axis.^29–34^ To prepare STO with mixed terminations, Nb-doped (0.5 wt %) STO was annealed at 1040 °C for 30 min in an oxygen atmosphere (9 × 10^–5^ Torr) in an ultrahigh vacuum (UHV) chamber. Three different measurements confirmed the termination of the resulting STO substrate: 1) *ex situ* lateral force microscopy (LFM) imaging under ambient conditions, 2) *in situ* atomic resolution STM imaging, and 3) tip–sample separation-dependent tunneling current (*I*/*Z* spectroscopy) measurements. As shown in Figure 1(b), the large-scale LFM image indicates that the as-prepared STO substrate has a step-and-terrace topography with wide and narrow terraces, exhibiting different friction contrasts. The high contrast terrace is attributed to the TiO_2_ termination, consistent with earlier LFM measurements showing higher friction on the TiO_2_ surface compared to the SrO-termination.^32^

**Figure 1:**
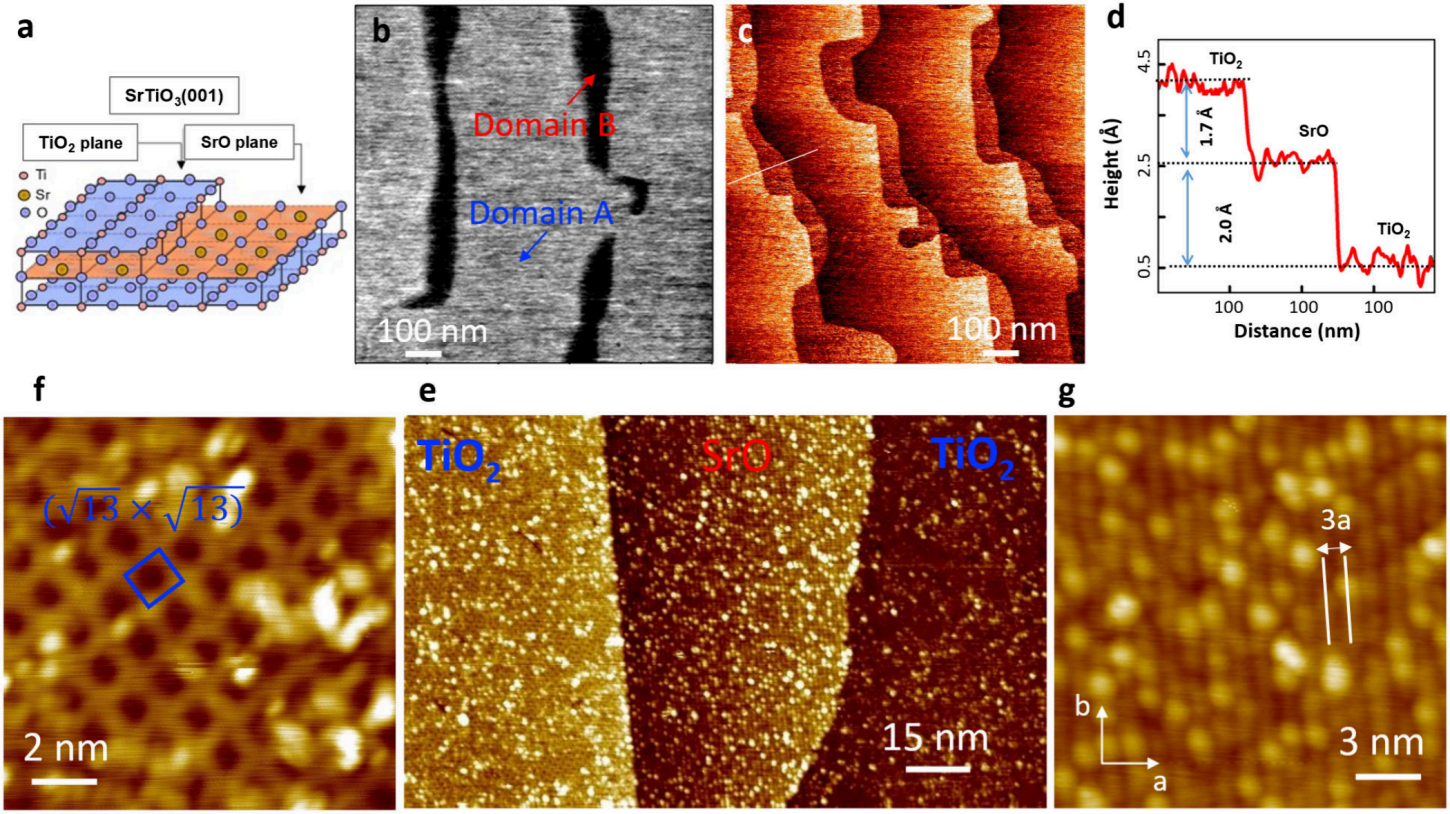
SrTiO_3_ (001) with mixed SrO and TiO_2_ terminations. (a) Schematic of SrTiO_3_(001) substrate with the TiO_2_ and SrO termination. LFM (b) and STM (c) images of the STO substrate with mixed TiO_2_ and SrO termination (*V*_s_ = 2.7 V, *I*_t_ = 0.1 nA). (d) Line profile along the white dashed line in (c). (e) STM image of the STO surface with coexisting TiO_2_ and SrO termination. (f) STM image of the TiO_2_ surface with the $\left(\sqrt{13}\times \sqrt{13}\right)$ reconstruction (*V*_s_ = 0.5 V, *I*_t_ = 0.1 nA). (g) STM image of the SrO surface with the (3 × 1) reconstruction (*V*_s_ = 2.0 V, *I*_t_ = 0.1 nA).

Such assignment of the surface termination is confirmed by *in situ* STM imaging, which also shows similar topography with alternating wide and narrow terraces separated by steps with the half-unit cell height of STO (Figure 1(c–e)). On the wide terrace, a $\left(\sqrt{13}\times \sqrt{13}\right)$ reconstruction is observed, consisting of a network of truncated octahedra TiO_5_ (Figure 1(f)), typical for the TiO_2_ termination.^34^ In contrast, the narrow terrace shows a row structure with a periodicity of ∼1.2 nm, three times the in-plane STO lattice constant *a*_STO_ (Figure 1(g)). Since no apparent periodicity is observed along the row, the structure is denoted (3 × 1), consistent with earlier work.^29,30,34^

Finally, the surface work function was determined by fitting the *I*/*Z* spectra [Supporting Information Figure S1], which yields barrier heights of 3.8 ± 0.3 eV and 4.7 ± 0.3 eV for the TiO_2_- and SrO-termination, respectively. The lowered surface work function of TiO_2_ termination is expected to facilitate higher charge transfer to FeSe from the STO substrate.^15,16^

Single-layer FeSe was grown on the mixed-terminated STO substrate at 350 °C, followed by annealing at temperatures up to 510 °C for 1–3 h. STM imaging shows that the FeSe films follow the STO substrate’s step-terrace topography (Figure 2(a)). On the same terrace, two domains of different contrasts and widths, separated by a boundary marked by the white dashed line, are identified as FeSe/TiO_2_ and FeSe/SrO based on atomic resolution imaging and differential conductance d*I*/d*V* measurements. The islands near the step edges with the highest contrast are the second layer FeSe, which is not superconducting.^35^ While the FeSe layer nearly covers the TiO_2_-terminated regions, pits are often observed to form on the SrO parts of the substrate. As shown in Figure S2­(a), for samples annealed for a longer time, while more than 90% of the SL FeSe has desorbed on the SrO termination, some part of the second layer FeSe remains on the TiO_2_ termination. The data suggest the coupling between FeSe and SrO surfaces is even weaker than between adjacent FeSe layers. The line profile shown in Figure S2 provides additional evidence to support these observations. The data indicate that the height of the SL FeSe on the SrO termination is around 8.7 Å, while the height on the TiO_2_ surface is only 6.1 Å. This smaller height of the FeSe/TiO_2_ interface suggests stronger interfacial coupling between the FeSe film and the TiO_2_ regions compared to the SrO regions.

**Figure 2:**
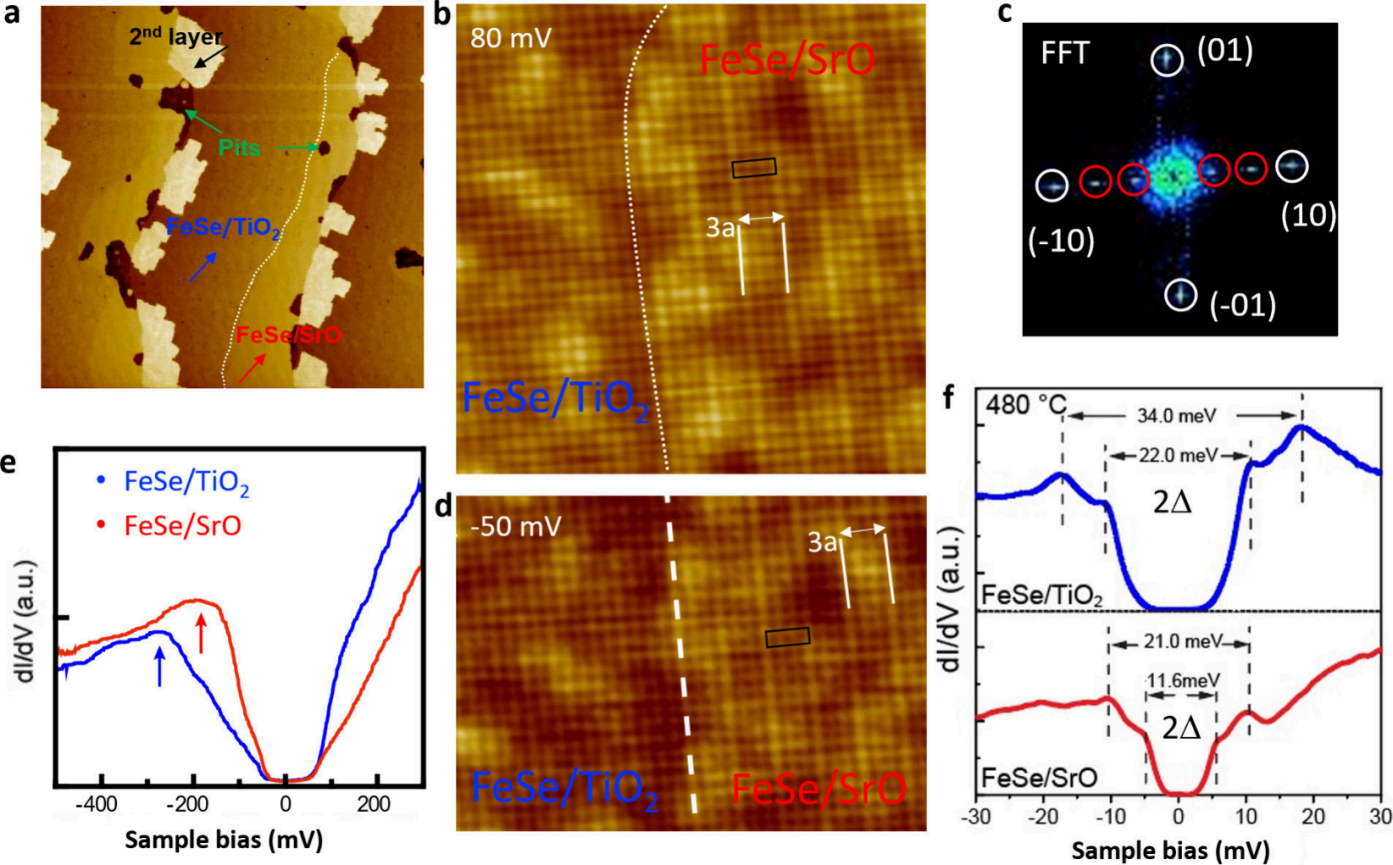
Superconducting properties of the single-layer FeSe/SrO and FeSe/TiO_2_. (a) Large-scale STM image of a SL FeSe film on STO with mixed TiO_2_ and SrO termination (*V*_s_ = 2.7 V, *I*_t_ = 0.1 nA). (b) Atomic resolution STM image of the SL FeSe/TiO_2_ and FeSe/SrO (*V*_s_ = 50 mV, *I*_t_ = 0.5 nA). The (3 × 1) unit cell is marked. (c) FFT analysis of the image in (b). (d) STM image of the SL FeSe/TiO_2_ and FeSe/SrO taken at a bias of −50 mV. The rectangle marks the (3 × 1) unit cell. (e) Representative d*I*/d*V* spectra of the SL FeSe/TiO_2_ and FeSe/SrO, and a close-up view near the Fermi level (f). The dashed lines mark the superconducting coherence peaks.

The atomic STM imaging further confirms the difference between FeSe/TiO_2_ and FeSe/SrO. While a square lattice is seen on both terminations, for SeFe/SrO, an additional stripe modulation is superimposed on the (1 × 1) lattice along the crystallographic *a*-axis. Fast Fourier transform (FFT) analysis indicates the modulation has a (3 × 1) periodicity. Interestingly, images acquired at energies below the Fermi level still exhibit the same periodicity and a similar contrast as those acquired above *E*_F_ (Figure 2(d)). These observations suggest that the initial (3 × 1) surface reconstruction of the SrO termination remains at the interface, consistent with the weaker interfacial coupling in FeSe/SrO, as discussed above. For the TiO_2_ termination, on the other hand, the original $\left(\sqrt{13}\times \sqrt{13}\right)$ structure is no longer visible in STM imaging, indicating stronger interfacial coupling in FeSe/TiO_2_.

The distinct electronic properties of the SL FeSe films on the two substrate terminations are revealed by comparing their d*I*/d*V* tunneling spectra, as shown in Figure 2(e). Specifically, the pronounced valence band peak is observed at −170 ± 20 meV for the FeSe/SrO interface, but it shifts downward to around −270 ± 20 meV for the FeSe/TiO_2_ interface. As this peak is associated with the degree of charge doping,^14^ the shift indicates higher electron doping in FeSe/TiO_2_, consistent with TiO_2_’s lower work function that favors electron transfer from the STO substrate.

To quantitatively determine the doping level, we compared the electronic structure of SL FeSe on the 100% TiO_2_- and mixed TiO_2_/SrO-terminations using ARPES. Figure 3(a) is an STM topographic image of the SL FeSe/TiO_2_/STO after annealing at 510 °C. Note that annealing is typically necessary to achieve superconductivity in the SL FeSe/STO films, often resulting in some FeSe desorption appearing as dark pits and white adsorbates (likely Fe clusters). Based on our DFT calculations (Supporting Information Figure S3), the Fermi surfaces of FeSe/TiO_2_ and FeSe/SrO are similar, consisting of two electron pockets at M, as confirmed by ARPES band maps. As shown in Figure 3(b,c), the band structure around M and Γ for FeSe/TiO_2_ shows electron pockets at M and no hole pockets at Γ, consistent with earlier work on superconducting FeSe/STO.^6,17^ For the SL FeSe on the mixed TiO_2_/SrO-termination (Figure 3(d)), the Fermi surface at M is similar to a slightly larger background, likely due to the higher number of pits present in the film. Nevertheless, it can still fit with two sets of eclipses, a smaller set attributed to the FeSe/SrO (Figure 3(e)) and a larger set attributed to the FeSe/TiO_2_ (Supporting Information Figure S4).

**Figure 3:**
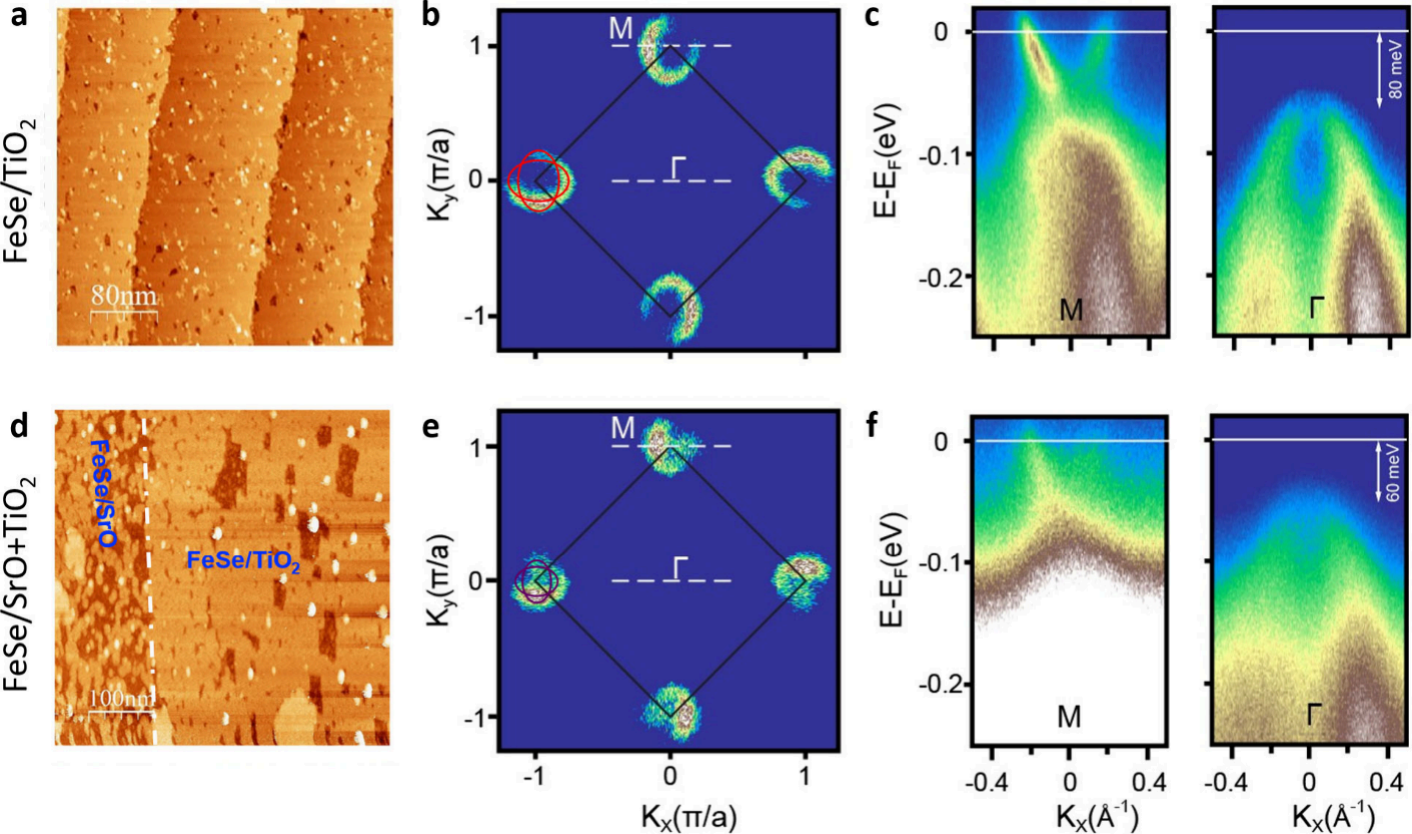
ARPES measurements of single-layer FeSe films on the single TiO_2_-terminated and mixed-terminated STO. (a–c) STM topographic image, Fermi surface map, and band structure around M and Γ of the SL FeSe on the STO substrate with 100% TiO_2_-termination. (d–f) Same as a–c, but on an insulating STO substrate with coexisting SrO and TiO_2_ termination. All measurements were conducted at 80 K.

Based on the size of electron pockets, the electron density is estimated to be (0.050 ± 0.005) e^–^ per Fe for the SL FeSe on mixed SrO/TiO_2_ termination, smaller than the (0.1000 ± 0.005) e^–^ per Fe on the TiO_2_ termination (Supporting Information Figure S5). This is consistent with the top of the hole band around the Γ point. For the FeSe/(SrO&TiO_2_), the band top is 60 meV below the Fermi level (Figure 3f), which is 20 meV closer to the Fermi level than that of FeSe/TiO_2_ (Figure 3(c)). Overall, ARPES measurements showed similar band structures in both cases, but with a 50% reduction in electron doping on the mixed SrO&TiO_2_ termination. This is consistent with larger interlayer spacing in the STM data for FeSe/SrO, and *I*–*Z* spectroscopy indicates a higher surface work function for the SrO termination, both unfavorable for charge transfer.

The intimate connection between the substrate termination and the electronic band structure and doping thus implies that the superconducting properties will likely be influenced significantly. To probe superconductivity, d*I*/d*V* spectra are obtained near the Fermi level (Figure 2(f)) (additional spectra in Supporting Information Figures S6–S7). The spectra are distinctly U-shaped with two pairs of coherence peaks, indicating that the FeSe films are superconducting on both terminations. However, the superconducting gap, defined as the separation between spectral peaks, exhibits a significant difference across the different regions. For the SL FeSe/TiO_2_, the gaps are measured to be 17.0 ± 0.5 meV and 11.0 ± 0.5 meV, similar to values reported in previous studies.^2^ In contrast, for the SL FeSe/SrO, the gaps are significantly lower at 10.5 ± 0.5 and 5.8 ± 0.5 meV. These results indicate a direct link between the superconducting gap and the substrate termination, which in turn controls the interfacial atomic structure.

To further investigate this connection, STEM measurements were carried out on superconducting SL FeSe grown on mixed-terminated STO substrates, capped by a 20-layer FeTe (Supporting Information Figures S8–S9). Figure 4(a,b) shows the high-angle annular dark field (HAADF) cross-sectional view of the FeSe/SrO and FeSe/TiO_2_ interfaces, overlaid with atomic structures of the FeTe capping layer, SL FeSe, and STO substrate. A significant difference between the two interfaces is an additional layer between FeSe and the double TiO_2_ layers in FeSe/TiO_2_. The nature of this additional layer is controversial, attributed to extra Se at the interface in some earlier STEM studies^36,37^ due to an energetically favorable Se O-vacancy complex^38^ and not observed in other studies.^39^ The discrepancy is likely due to differences in postgrowth annealing conditions, which are essential for inducing superconductivity.^37^ Our energy dispersive spectroscopy (EDS) analysis indicates that this additional layer is indeed Se, labeled as Se_3_ in Figure 4(d). Details of the EDS atomic profiles of the K and L edges of Fe, Se, Ti, and Sr are shown in Figure S10, which verify that the FeSe/TiO_2_ interface contains double TiO_2_ (Ti_1_ and Ti_2_) and additional Se (Se_3_) layers.

**Figure 4:**
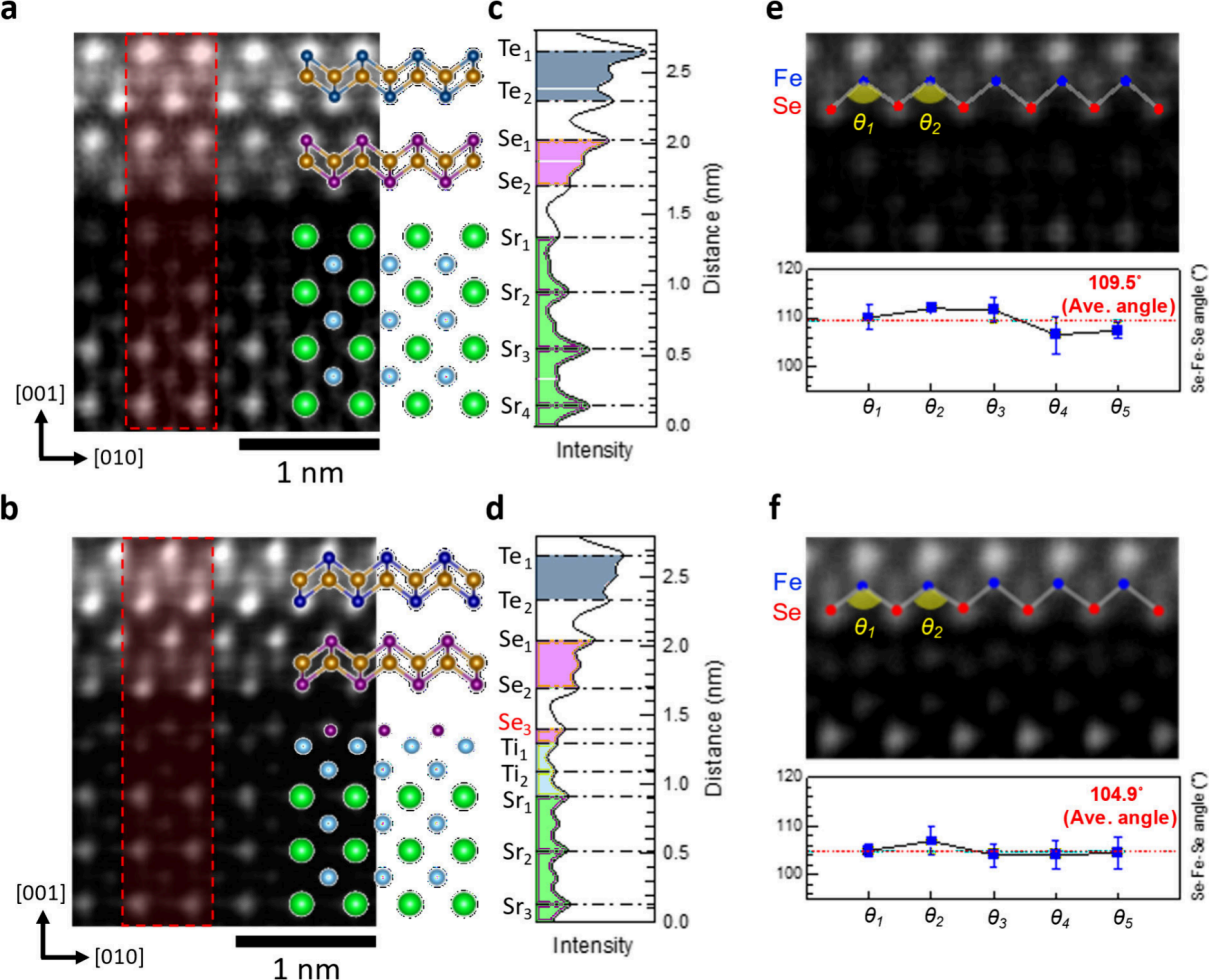
STEM imaging of single-layer FeSe on SrO- and TiO_2_-terminated STO substrates. (a,b) Cross-sectional HAADF-STEM images showing the interface structures of the SL FeSe/SrO and FeSe/TiO_2_, respectively. (c,d) Intensity profiles along [001] from the areas indicated by dotted boxes in (a) and (b). (e,f) The measured Se–Fe–Se buckling angles for the SL FeSe at the SrO and TiO_2_ interfaces, respectively. The error bars represent the standard deviation calculated from multiple measurements taken across different regions. The buckling angle of the FeSe/TiO_2_ is smaller by ∼5 degrees than that of the FeSe/SrO.

In addition, a close examination of the HAADF-filter images of the interfaces reveals that the average Se–Fe–Se angle is 104.9° and 109.5° for the FeSe/TiO_2_ and FeSe/SrO interfaces, respectively (Figure 4(e,f), additional data in Supporting Information Figure S11). The angle for the FeSe/TiO_2_ termination is comparable to that reported in an earlier study.^40^ Those STEM results confirm distinctive interfacial atomic-scale structures and FeSe tetrahedral geometry between the SL FeSe on TiO_2_ and SrO. While the effect of optimal Se–Fe–Se bond angle on superconductivity is recognized in earlier work,^41^ direct comparison of interfacial structures between different studies is challenging due to different annealing conditions used, as noted in recent work.^25,37^ Here, the growth of FeSe on a mixed-terminated STO substrate enables us to directly compare the effect of Se–Fe–Se bond angles on superconductivity.

The epitaxial growth on TiO_2_- and SrO-terminated STO modifies the SL FeSe properties in two significant ways: 1) it changes the surface work function that controls charge transfer and electron doping, and 2) it causes distortion of the FeSe tetrahedron and changes the Fe–Se–Fe bond angle that, in turn, controls electron correlations.^28^ The larger pairing gap on TiO_2_ termination is consistent with its smaller work function, which favors charge transfer.^15,16^ However, recent work on the FeSe/FeO_*x*_ interface has shown that higher *T*_C_ can be achieved with a lower doping level.^26,27^ This indicates that, in addition to electron doping, other factors can also play a significant role. The bulk STO phonon contributes equally to the electron–phonon coupling for both surface terminations; therefore, they cannot account for the observed difference in pairing gaps. Nevertheless, the localized interfacial phonon may still play a significant role, as revealed by recent studies on the FeSe/TiO_2_ interface.^24,25^ Similar studies are needed to explore the role of localized phonons at the FeSe/SrO interface. Alternatively, electron correlations, which are known to enhance electron–phonon coupling in bulk FeSe,^42,43^ may also contribute, as we discuss below for the SL FeSe/STO systems.

To explain the phenomenological connection between the superconducting gap and bond angle, we calculated the atomic-scale geometry of FeSe on STO with different terminations by eDMFT (Supporting Information Figure S12). Previously, it was found that the O-vacancy in STO is responsible for (i) donating electrons to FeSe^28,44^ and (ii) controlling the Se–Fe–Se angle.^28^ Here, we considered a (1 × 1) unit cell with 50% oxygen vacancies in the TiO_2_ termination and 100% or 0% O-vacancy for the SrO termination. The computed Se–Fe–Se angle differs significantly. For 0% O-vacant SrO, the angle is 110.4°, close to the free-standing FeSe.^28^ For 100% O-vacancy SrO-termination, it becomes 106.8°. For double TiO_2_ termination with 50% O vacancies, the angle is 107.4°. Thus, the Se–Fe–Se angles measured from STEM are closer to 0% O-vacancy on the SrO termination and 50% O-vacancy on the double TiO_2_ termination. This changes the strength of the electron correlations in this system as it is directly related to the Se–Fe–Se angle.^28^

To quantify this effect, we computed the orbital-dependent spectral weights *Z* on the self-energy obtained directly from the imaginary frequency from the continuous time Monte Carlo (CTQMC) simulations, which are shown in Figure S12­(d) for four different structures: (1) SL FeSe on the single TiO_2_ termination without O-vacancy, and (2) with 50% O-vacancy, (3) SL FeSe on the double TiO_2_ termination with 50% O-vacancy, and (4) SL FeSe on the SrO termination without O-vacancy. The results show that the Se–Fe–Se angle controls the strength of the correlations, and the d_*xy*_-orbital has the strongest correlations in all cases, a trend similar to that predicted for the single TiO_2_-terminated STO.^28^ Irrespective of the substrate termination, the system becomes more correlated with the O-vacancy than those without the O-vacancy. On the other hand, the change in Z is minimal from the 1TiO_2_ to 2TiO_2_ termination but significant when the STO is terminated with SrO, as a result of the change in the Se–Fe–Se angle. For FeSe/2TiO_2_ with O-vacancy, the angle is close to the “optimal angle” of 107°, resulting in the strongest correlations for all five d-orbitals. These results indicate that the smaller gap on the SL FeSe/SrO-terminated STO is likely due to the increase in the Se–Fe–Se angle, which weakens the correlations.

The observed trend of higher *T*_C_ on the TiO_2_ termination, accompanied by a smaller Se–Fe–Se tetrahedral angle, is similar to that found in bulk FeSe, where the angle, and thus the *T*_C_, is also tunable by applied pressure and intercalation of cationic spacer layers. At ambient pressure, the *T*_C_ of bulk FeSe is 8 K^8^ with a superconducting gap of Δ = 2.2 meV^45^ and a Se–Fe–Se angle of ∼104°.^46^ At an applied pressure of 7 GPa, the *T*_C_ increases to 37 K, with a decreased bond angle of ∼102°.^47,48^ Similarly, the intercalation of a Li_*x*_(NH_2_)_*y*_(NH_3_)_1–*y*_ spacer layer increases the *T*_C_ to 43 K, with increased interlayer spacing and Se–Fe–Se bond angle of 102.9°.^49^ Interestingly, for alkaline metal intercalated FeSe, a modest enhancement in *T*_C_ to 30 K is observed.^50,51^ For the intercalated system, interlayer spacing is another variable that controls electron screening, as suggested by recent calculations that have shown decreasing bond angle reduces hopping, while increasing the *c*-axis reduces electronic screening, both of which could enhance electron correlations and increase *T*_C_.^52,53^ For the single-layer FeSe/STO, variations in the interlayer spacing between the substrate surface and the FeSe film, as observed here, can modulate the strength of interfacial EPC, thereby affecting *T*_C_.^17,25^ Moreover, our recent work shows that optimal electronic correlations can further enhance the EPC within the FeSe film itself,^54^ motivating future studies of the substrate termination effect on the EPC of the film and that of the interface.

In conclusion, the epitaxial growth of single-layer FeSe on SrTiO_3_(001) substrates with coexisting TiO_2_ and SrO terminations provides a unique platform for identifying the key factor driving high-temperature superconductivity. For single-layer FeSe/TiO_2_, we observed a 60% larger superconducting gap, a distinct interfacial structure with a smaller Se–Fe–Se angle, and stronger electron correlations. By independently tuning the surface work function and the FeSe tetrahedral geometry, our results demonstrate that electron correlations are optimal in FeSe/TiO_2_ for achieving higher *T*_C_ through either electronic or phononic origin. These findings highlight the potential of fusion 2D materials with correlated complex oxide substrates through designed interfaces to control quantum phases via interfacial charge transfer doping, electron–phonon coupling, electron correlations, and light–matter interactions.

## Supplementary Material



## Notes

Methods, supplementary notes on eDMFT and DFT calculations, supplementary Figures 1–12 (spatially resolved *I*–*z* spectra on the TiO_2_ and SrO termination, STM image and line-profile across a boundary between FeSe/TiO_2_ and FeSe/SrO, DFT bands, ARPES Fermi surface maps, doping level calculations, spatially resolved d*I*/d*V* spectra near the Fermi level on FeSe/TiO_2_ and FeSe/SrO, STM image of the FeTe capping layer, TEM sample preparation, STEM-EDS analysis of single-layer FeSe on a TiO_2_-terminated STO substrate, Se–Fe–Se bond angle analysis across multiple regions, eDMFT calculated atomic and band structures of the single-layer FeSe on TiO_2_- and SrO-termination STO) (PDF)
