# Supplementary material for: Unraveling Enhanced Superconductivity in Single-Layer FeSe through Substrate Surface Terminations
Source: Nano Lett. 2025 Sep 3;25(47):16572–9. doi: 10.1021/acs.nanolett.5c01298 (PMC12670505; doi:10.1021/acs.nanolett.5c01298)
Supplement: Supplementary file 1 [file nl5c01298_si_001.pdf]

Supplementary Information

Unraveling Enhanced Superconductivity in Single-layer FeSe through Substrate Surface Terminations

Qiang Zou[1], Gi-Yeop Kim[2], Jong-Hoon Kang[3], Basu Dev Oli[1], Zhuozhi Ge[1], Michael Weinert[4], Subhasish Mandal[1], Chang-Beom Eom[3], Si-Young Choi[2,5], and Lian Li[1,*]

[1]Department of Physics and Astronomy, West Virginia University, Morgantown, WV 26506, USA

[2]Department of Materials Science and Engineering, Pohang University of Science and Technology, Pohang 37673, Republic of Korea

[3]Department of Materials Science and Engineering, University of Wisconsin-Madison, Madison, WI 53706, USA

[4]Department of Physics, University of Wisconsin, Milwaukee, WI 53211, USA

[5]Center for Van der Waals Quantum Solids Institute for Basic Science, Pohang 37673, Republic of Korea

Methods:

Sample preparation: To create both TiO_2 and SrO terminations, Nb-doped (0.5% wt) $SrTiO_3$ (001) was treated under an oxygen atmosphere in an ultra-high vacuum (UHV) chamber with the base pressure < 1.0×10^{-9} Torr. The substrates were first degassed at 500 °C – 600 °C for one hour and then annealed at 1040 °C for half an hour with the $P_{O2} = 9.0 \times 10^{-5}$ Torr. After that, the STO substrates were transferred into the molecular beam epitaxy (MBE) chamber with a base pressure better than 3.0×10^{-10} Torr. The single-layer FeSe films were grown via co-evaporating ultrahigh-purity Se (99.999%) and Fe (99.995%) with a flux ratio of ~10:1 on the heated STO substrates at a temperature of ~400 °C. The growth rate of FeSe films is dominated by the Fe flux, which is around 0.5 monolayers per minute. The as-grown FeSe films were annealed at ~ 420, 480, and 510 °C for up to three hours.

STM/S: The measurements were performed at 4.5 K in ultrahigh vacuum low-temperature STM. Polycrystalline PtIr tips were used and tested on Ag/Si(111) films before the STM/S measurements. dI/dV tunneling spectra were acquired using a standard lock-in technique with a small bias modulation V_{mod} (2% of the setting point bias) at 732 Hz. All I/Z spectra were conducted at setting 3 V and 100 pA with feedback off.

ARPES: ARPES was carried out at 80 K with a Scienta DA30 analyzer and He discharge lamp (hv = 21.218 eV). The energy resolution was set at ~9 meV, and the angular resolution was 0.3°.

STEM: Superconducting single-layer FeSe films on mixed-terminated STO substrates were protected by a 20-layer FeTe before STEM imaging. For cross-sectional analysis on interfacial structure, samples were prepared using a dual-beam focused ion beam system (Helios G3, Thermo Fisher Co., USA) along the [010] projection. STEM analyses were performed with an aberration-corrected STEM (JEM-ARM200F, JEOL, Japan) at 200 kV equipped with a fifth-order spherical aberration corrector (ASCOR, CEOS GmbH, Germany) at the Materials Imaging & Analysis Center of POSTECH. The electron probe size was set to approximately 70 pm, and the collection semi-angle ranged from 54 to 216 mrad for high-angle annular dark-field (HAADF) imaging. The raw STEM data were filtered using a Local 2D Difference Filter (Filters Pro, HREM Research Inc., Japan) to reduce background scanning noise without introducing structural distortions. Multiple regions across the sample were analyzed to extract Se-Fe-Se bond angles. The bond angles were determined by identifying atomic positions through Gaussian fitting of atomic intensities, followed by angle calculation between adjacent Se-Fe-Se atomic triplets. Image analysis was conducted using Python with customized atomic position analysis codes.

STEM-EDS: Atomic-scale chemical analysis was conducted using energy-dispersive X-ray spectroscopy (EDS) performed with dual silicon drift detectors (JED-2300T, JEOL) attached to the STEM system. EDS line profiles were acquired along the [001] direction in the same regions used for STEM imaging.

Spatial broadening in EDS mapping: On the microscope used for this study (JEOL ARM200F (200 kV)), the nominal probe size in high-resolution STEM imaging (9C) is approximately 0.08–0.10 nm. However, for STEM-EDS acquisition, a 7C probe condition was used to increase the X-ray count rate, which corresponds to a nominal probe size of approximately 0.2–0.3 nm. While the probe size in EDS mode was indeed larger than in imaging mode, the observed Sr signal tail shown in Fig. S10 is not primarily caused by the larger probe diameter. In EDS mapping, the effective spatial resolution is dominated by other factors, including:

1. X-ray generation volume within the specimen
2. Specimen thickness and electron beam channeling along atomic columns
3. Delocalization of X-ray emission into adjacent atomic planes

These effects can cause the apparent elemental signal to extend 1 nm or more laterally or along the beam direction, even when using a sub-angstrom probe. This spatial broadening explains the weak Sr signal in the top TiO_2 bilayer (Fig. S10) as a tailing artifact, rather than evidence of actual Sr intermixing. This interpretation is consistent with Ref. 37 (PRB 105, 165407 (2022)), which reports resolution-limited Sr/Ti signal tails extending into adjacent planes due to probe-induced delocalization.

Supplementary Notes

eDMFT calculations: We used the embedded implementation of the all-electron DFT+dynamical mean field theory (DMFT) method, which has been successful in describing the electronic structure of Fe-based superconductors [Ref: SM1, SM2, SM3, SM4]. eDMFT functional is defined in real space and implemented in the very accurate all-electron linear augmented plane wave (LAPW) basis, as implemented in Wien2k[RefSM5]. The quantum impurity method is solved by the continuous-time quantum Monte Carlo method (CTQMC) [Ref: SM6]. The values of Hubbard U and Hund's J were set to 5 and 0.7 eV, respectively.

Previously, from eDMFT computations, it was found that O-vacancy in STO is responsible for i) donating electrons to single-layer (SL) FeSe, and ii) controlling the Se-Fe-Se angle (Ref. [28]). Here, we consider a (1x1) unit cell with 50% oxygen vacancy in the TiO_2 termination and 100% and 0% O-vacancies for the SrO termination.

Figure S11 shows the schematic representation of FeSe/STO heterostructures for single TiO_2, double TiO_2- and SrO-terminated STO. We optimized the atom positions using eDMFT and found that the O-vacancy predominantly controls the height of the SL FeSe film from the substrate. The unique feature in the eDMFT implementation for computing forces and thereby obtaining precise atom positions is essential here as the Se-Fe-Se angle is severely underestimated in standard density functional theory calculations, especially in this paramagnetic phase. From the eDMFT optimization, we found that the height between film to substrate increased from 2.88 Å in TiO_2 termination to 3.4 Å for the SrO-terminated STO without O-vacancy. In comparison, the height decreased to 2.53 Å for SrO termination with 100% O-vacancy. This height is crucial for controlling the amount of electron doping from the substrate to the FeSe film, which is directly observed in our ARPES and STS measurements. Since the amount of O-vacancy is not directly available from the experiment [Ref:SM7], we consider 0% and 100% O-vacancy in a 1x1 SrO-terminated STO in our computations.

Next, we note a change in the computed Se-Fe-Se angle in these two terminations. Since the FeSe layer is away from the substrate for 0% O-vacant SrO, it becomes close to the free-standing FeSe layer's geometry, where the angle is close to 110.4 degrees [28]. For double TiO_2 termination, this angle is close to 107.4 degrees. For SrO-terminated STO with O-vacancy, the Se-Fe-Se angle becomes 106.8 degrees. The Se-Fe-Se angles measured from STEM are closer to 0% O-vacancy in SrO termination and 50% O-vacancy in double TiO_2 termination. This changes the strength of the electron correlation in this system as it is directly related to the Se-Fe-Se angle, as was seen in Ref. [28].

Next, we describe eDMFT computed orbital-resolved spectral functions for SrO and TiO_2 terminations (Figs. S11(b,c)). The electron and hole pockets are mainly made of d_{xy} (red) and d_{xz+yz} orbitals (green). With 0% O-vacancy SrO-terminated STO, we notice that the outermost d_{xy} (red) hole pocket moves down in energy while the inner d_{xz+yz} pockets move up compared to the TiO_2-terminated STO. The central hole pocket almost degenerates in the 0% O-vacancy SrO-terminated STO.

To quantify the structure tuning effect through the change in termination on the electron correlations, we computed the orbital-dependent spectral weights Z (inverse of mass enhancement) on the self-energy obtained directly from the imaginary frequency (Fig. S11(d)) from the continuous time Monte Carlo (CTQMC) simulations. Here, we compute Z for four different structures, namely (1) FeSe on single TiO_2-terminated STO without (FeSe/1TiO_2-NoVac) and (2) with O-vacancy (FeSe/1TiO_2-OVac), (3) SL FeSe on double TiO_2-terminated STO with O-vacancy FeSe/2TiO_2-OVac), and (4) SL FeSe on SrO terminated STO without O-vacancy (FeSe/SrO-NoVac). We notice that the trend is strikingly similar to that predicted in Ref. [28] for a single TiO_2-terminated STO. We also see that the angle controls the strength of the correlations. The d_{xy}-orbital has the strongest correlations in all these cases.

Since the oxygen vacancy in STO changes the Se-Fe-Se angle, it plays an important role irrespective of substrate termination. The systems become more correlated with O-vacancy in TiO_2 or SrO termination than those without O-vacancy. On the other hand, the Z change is minimal from 1TiO_2 to 2TiO_2 termination but significant when the STO is terminated with SrO, as a result of the change in the Se-Fe-Se angle. For example, for either FeSe/1TiO_2-OVac or FeSe/2TiO_2-OVac, the angle is close to the 'optimal angle' of 107 degrees, which has the strongest correlation for all five orbitals, whereas for FeSe/SrO-NoVac the angle is 110.5 deg and the system becomes less correlated. These results indicate that the reduction of the gap in the SrO-terminated STO is due to the change in the Se-Fe-Se angle, which weakens the correlations. Overall, the trend in the computed Z with Se-Fe-Se angle explains the change of the measured gap with different terminations in STO.

DFT calculations: The FeSe/STO interfaces were modeled using symmetric slabs of (1x1) STO terminated by either double TiO_2 or SrO layers to avoid spurious dipoles between the repeated images. The calculations were carried out using VASP, including spin-orbit coupling and U=0.1 eV on the Fe sites, and assumed checkerboard magnetic order on the FeSe. The interface structure was relaxed, including the DFT-D3 with Becke damping van der Waals correction. The calculated nominal Fe-substrate separations and θ values for the different terminations and registries varied by more than 1 Å

and between ~109-111°, respectively, due to the different possible bonding between the substrate and the interface Se in particular.

As seen in the figure, the placement of the Fermi level in the calculations is very sensitive, at the level of 10's of meV, to details of the calculation and the interface: The choice of symmetric slabs means that the substrate is not stoichiometric (although there is a band gap). The calculations do not include the Nb doping nor possible oxygen vacancies and always assume charge neutrality of the whole system. Changes in the position of the Fermi level within the STO band gap mean that the overlap between the valence (bonding) states of FeSe and STO states. An additional complication is the assumed checkerboard AFM configuration, while it is known that FeSe does not order magnetically; as shown previously, fluctuations will broaden out the states in the spectra [SM8]. Given these caveats, the dispersion of the bands around M for the different interfaces is very similar, implying that the Fermi surface area of the M pocket provides a direct measure of the relative doping levels.

References:

SM1: S. Mandal, R. E. Cohen, K. Haule, Pressure suppression of electron correlation in the collapsed tetragonal phase of $CaFe_2As_2$: A DFT-DMFT investigation. *Physical Review B* **90**, 060501 (2014).

SM2: Mandal, S., Haule, K., Rabe, K.M. *et al.* Electronic correlation in nearly free electron metals with beyond-DFT methods. *npj Comput Mater* **8**, 181 (2022). https://doi.org/10.1038/s41524-022-00867-8

SM3: S. Mandal, R. E. Cohen, K. Haule, Valence and spin fluctuations in the Mn-doped ferroelectric $BaTiO_3$. *Physical Review B* **98**, 075155 (2018).

SM4: S. Mandal and R. Pati, Mechanism behind the switching of current induced by a gate field in a semiconducting nanowire junction, Phys. Rev. B **84**, 115306 (2011); https://doi.org/10.1103/PhysRevB.84.115306

SM5: P. Blaha, K. Schwarz, G. Madsen, D. Kvasnicka, J. Luitz, WIEN2k: An Augmented Plane Wave plus Local Orbitals Program for Calculating Crystal Properties. *Technische Universität Wien, Wien* **28**, (2001).

SM6: K. Haule, Quantum Monte Carlo impurity solver for cluster dynamical mean-field theory and electronic structure calculations with adjustable cluster base. *Physical Review B* **75**, 155113 (2007).

SM7: Dagdeviren *et al.* Length Scale and Dimensionality of Defects in Epitaxial SnTe Topological Crystalline Insulator Films, Adv. Mater. Interfaces, **4**, (2017) https://doi.org/10.1002/admi.201601011.

SM 8: T. Shishidou, D. F. Agterberg & M. Weinert, Magnetic fluctuations in single-layer FeSe, Communi. Phys. **1**, 8 (2018).

Supplementary Figures S1-10

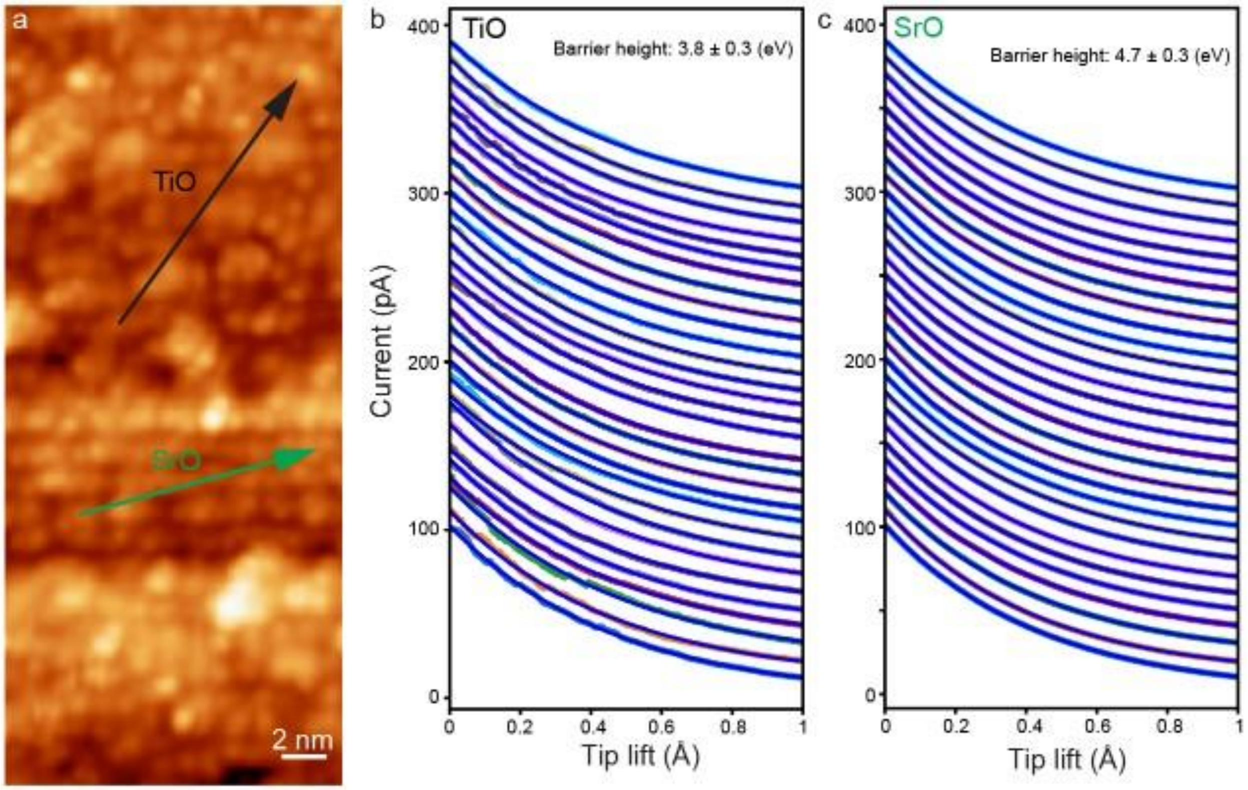

Figure S1 | Spatially resolved I-z spectra on TiO_2 and SrO termination. a, STM image of coexisting TiO_2 and SrO termination. b&c, spatially resolved I-z spectra taken along the two arrows in (a).

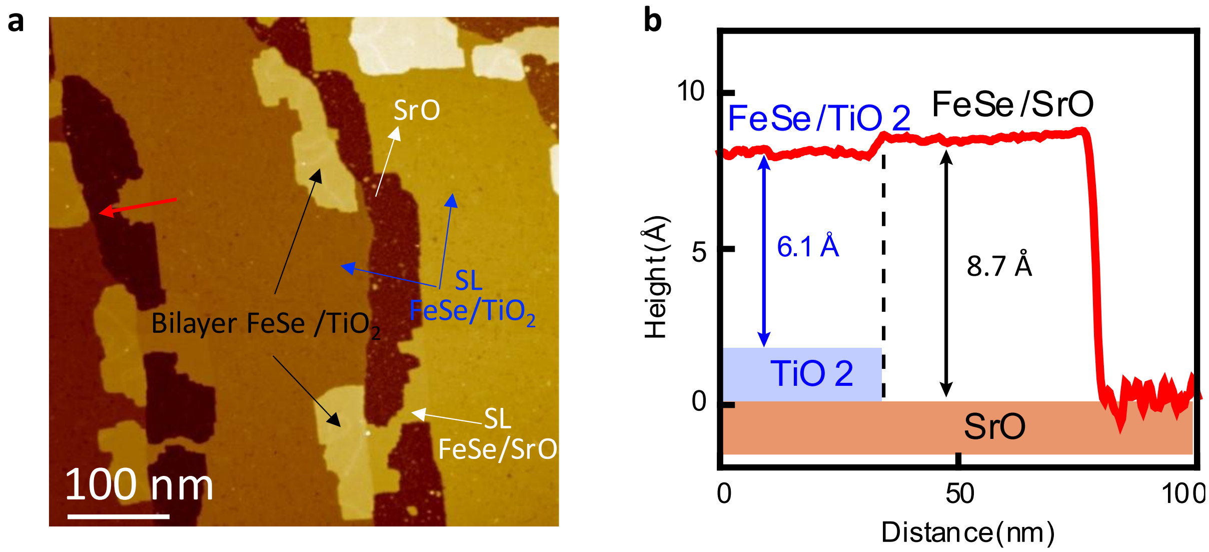

Figure S2 | (a) STM image of the SL FeSe on STO with mixed termination. (b) Line profile along the red arrow in (a).

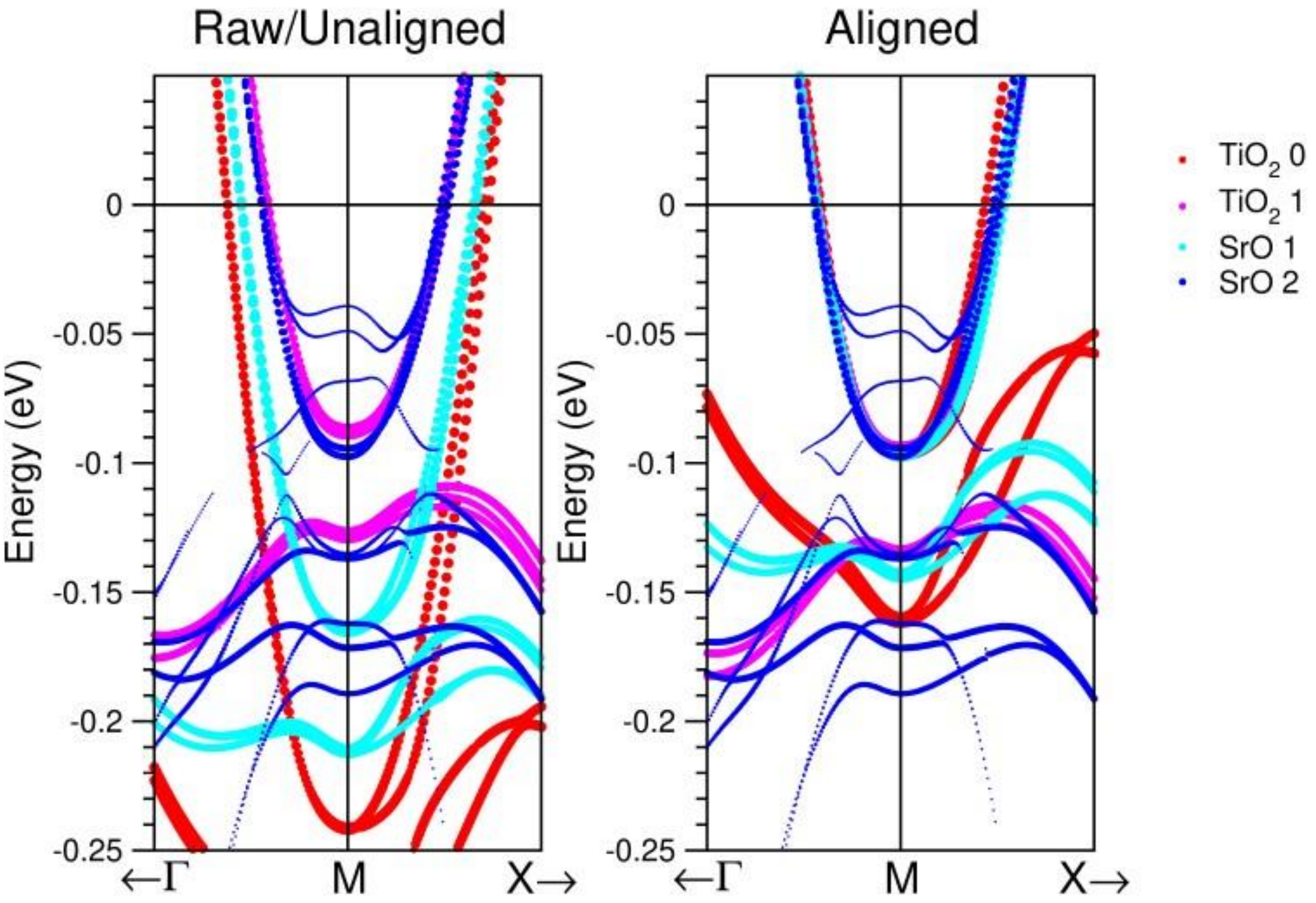

Figure S3 | Projected DFT band structures for the SL FeSe/STO ±0.4 Å-1 around M towards Γ and X for 2 registries of both the double TiO_2 and SrO terminations, as calculated (left panel) and shifted (right panel) to align the M point states. The size of the dots corresponds to the weight of the state (> 5%) on the FeSe layer.

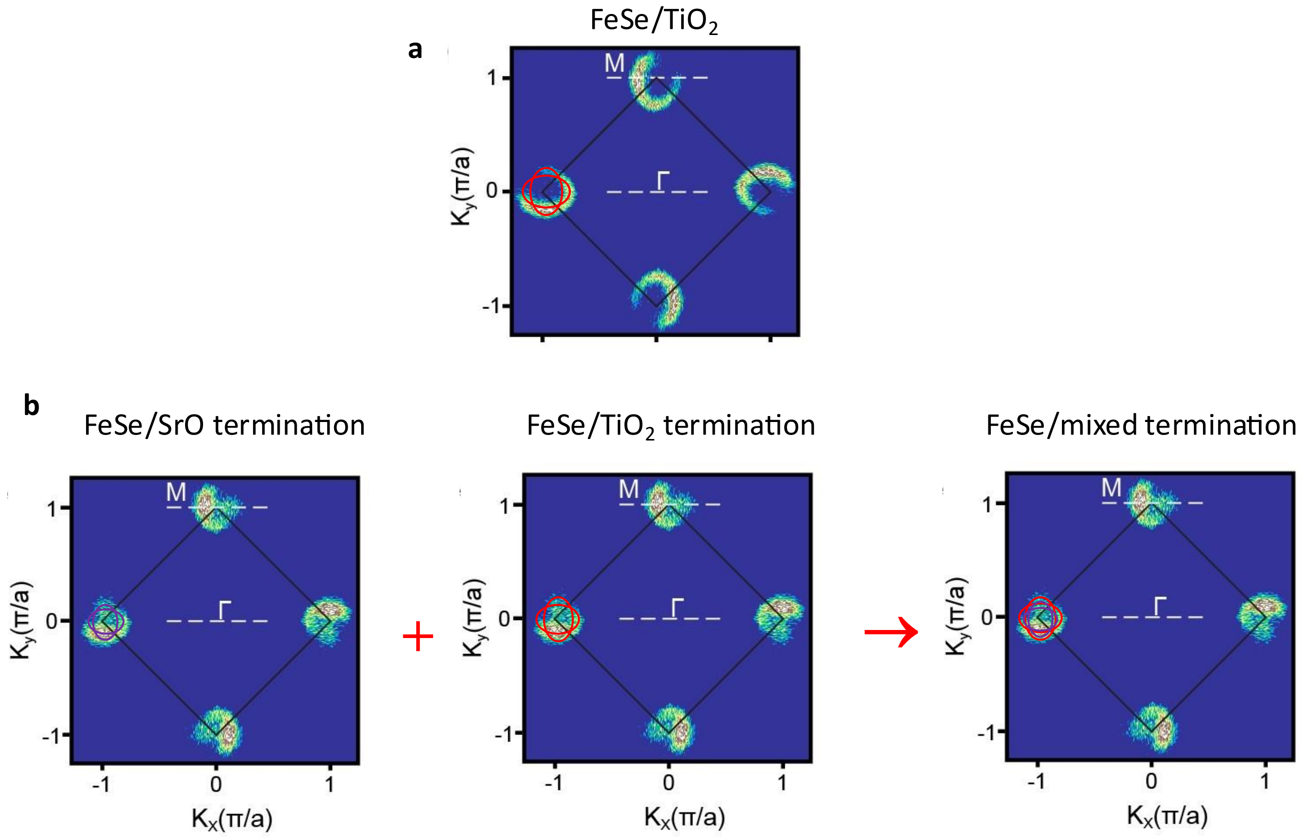

Figure S4 | ARPES map of the Fermi surface of the SL FeSe/STO. a, FeSe/TiO_2-STO, consisting of two eclipses. b, Similarly, the Fermi surface of FeSe/mixed termination: FeSe/SrO + FeSe/TiO_2.

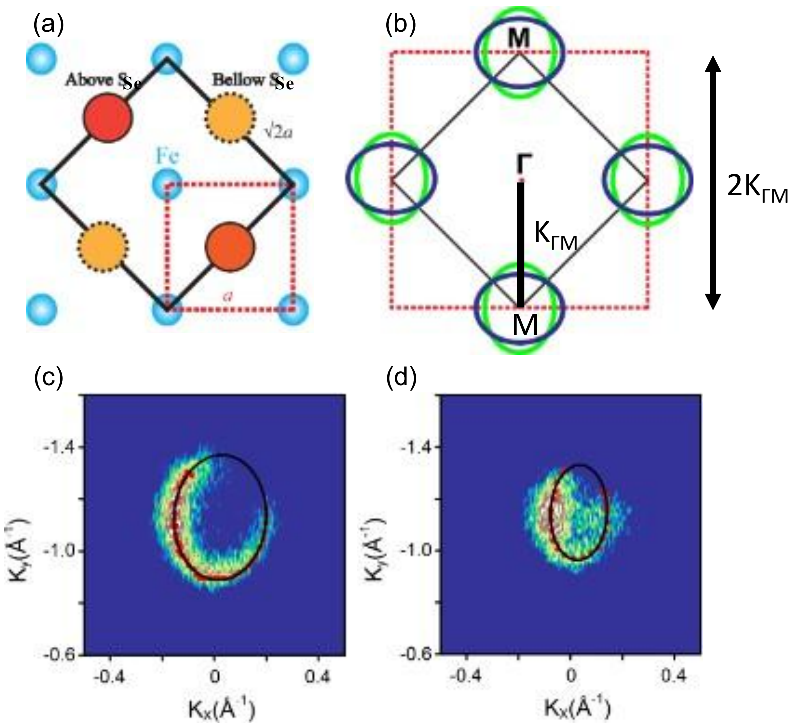

- Size of the 1st Brillouin zone of one Fe: $S_{BZ} = (2K_{\Gamma M})^2$.

- Size of the Fermi surface in the 1st Brillouin zone of one Fe:
  $S_{FS} = (4\ S_{green} + 4\ S_{blue})/2 = (2\ S_{green} + 2\ S_{blue}) = 4\ S_{green}$.

- Carrier density $= S_{FS} / S_{BZ} = 4\ S_{green} / (2K_{\Gamma M})^2 = S_{green} / K_{\Gamma M}^2$.

Figure S5 | Ball-and-stick model of one-Fe versus two-Fe lattice unit cell (a) and Brillouin Zone (b) of the SL FeSe (2 Fe unit cell). Fermi surface fitting of the SL FeSe on 100% TiO_2-terminated (c) and mix-terminated (d) STO.

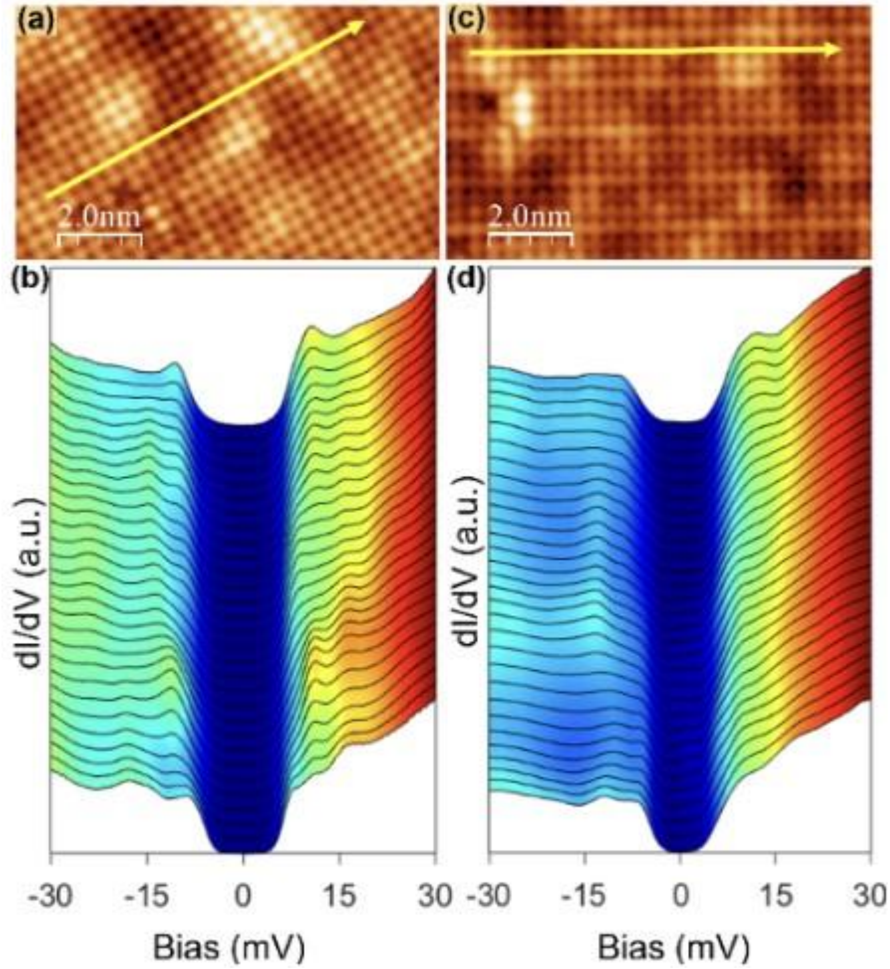

Figure S6 | Spatially resolved dI/dV spectra on FeSe/TiO_2 and FeSe/SrO. (a, c) STM images of FeSe/TiO_2 and FeSe/SrO (top panels). (b, d) Spatially resolved dI/dV spectra taken along the two arrows marked in the top panels.

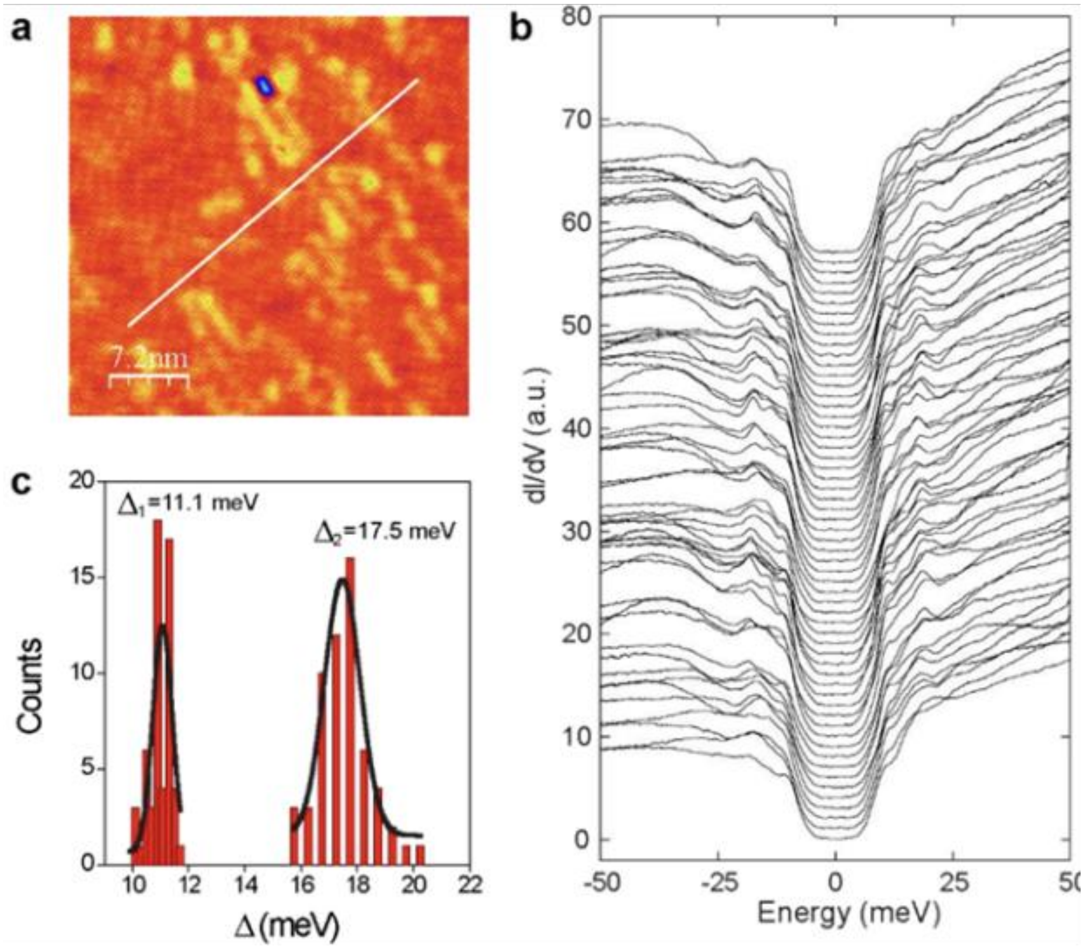

Figure S7 | (a) STM image of a typical SL FeSe/100% TiO_2-STO. (b) Spatially resolved dI/dV spectra taken along the white line in (a). (c) Superconducting gap distributions.

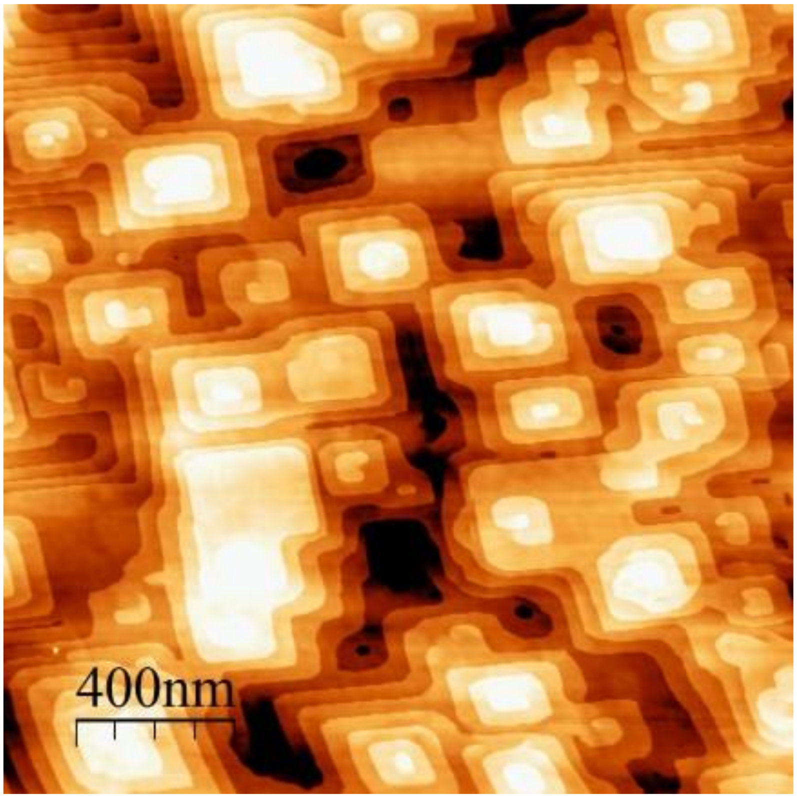

Figure S8 | STM image of the FeTe capping layer (~20 SLs).

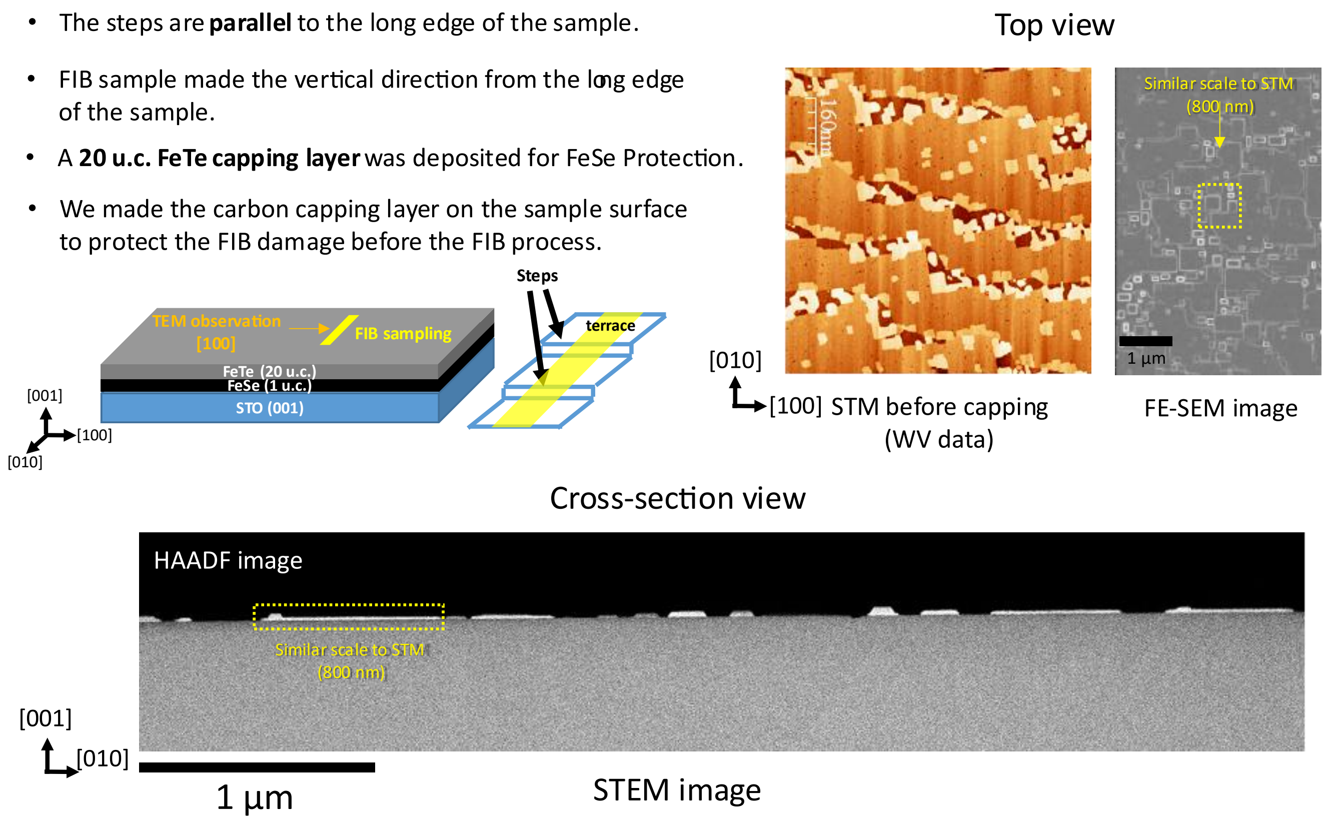

Figure S9 | TEM sample information and large field of view image.

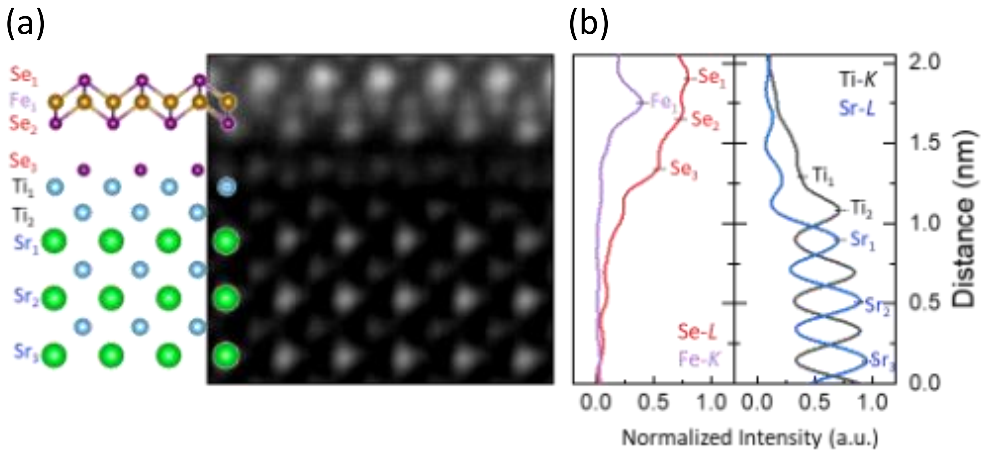

Figure S10 | STEM-EDS analysis of single-layer FeSe on a TiO_2-terminated STO substrate. (a) Cross-sectional HAADF-STEM image with atomic structures overlaid at the FeSe/TiO_2 interface. (b) The EDS atomic profile corresponds to the HAADF image in (a), showing that the FeSe/TiO_2 interface contains double TiO_2 (Ti1 and Ti2) and additional Se (Se3) layers.

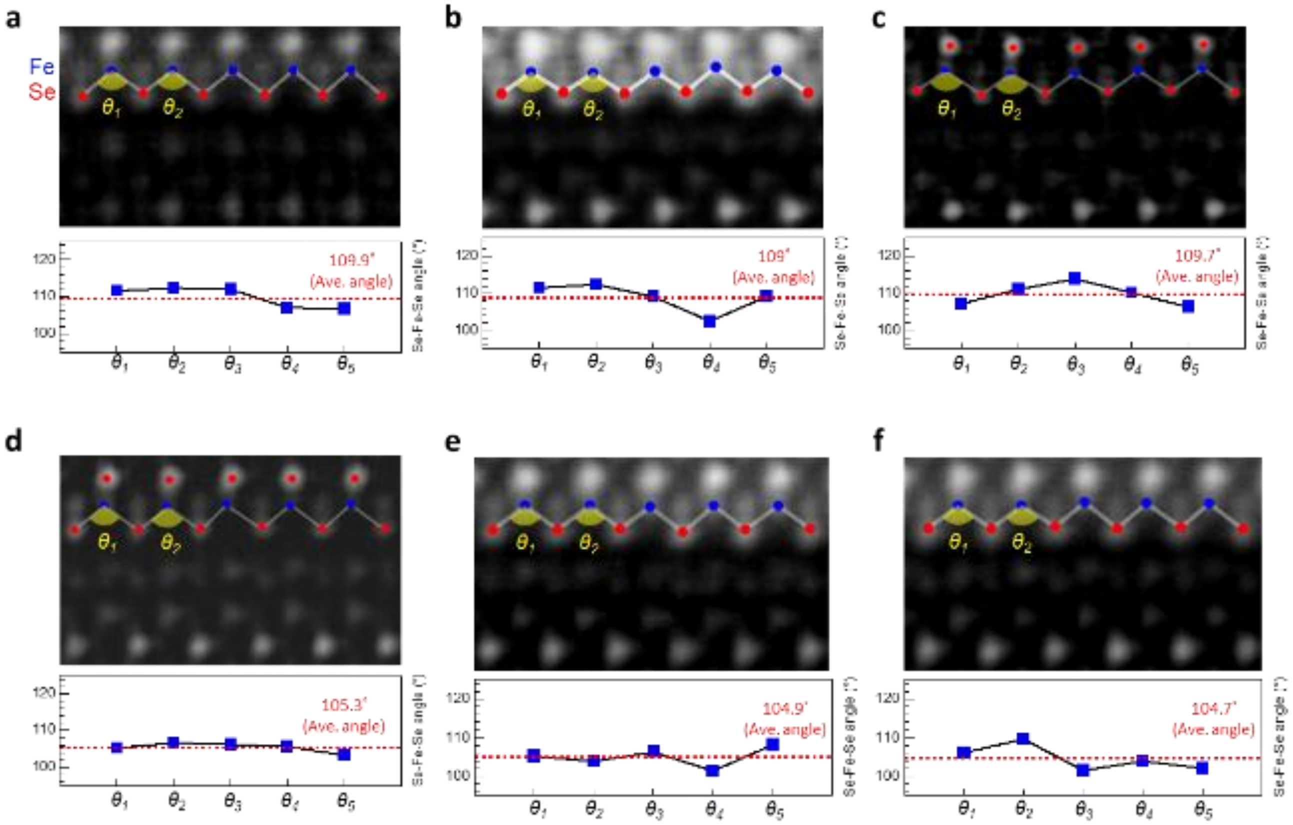

Figure S11 | Se-Fe-Se bond angle analysis across multiple regions. (a–c) Representative cross-sectional HAADF-STEM images and corresponding Se-Fe-Se bond angles measured across different regions of the FeSe/SrO interface. (d–f) Representative cross-sectional HAADF-STEM images and corresponding Se-Fe-Se bond angles measured across different regions of the FeSe/TiO_2 interface. For each region, the measured bond angles at specific Se-Fe-Se triplets (θ_1–θ_5) are plotted along with the average bond angle (red dashed line). These measurements demonstrate the reproducibility of the bond angle differences observed between the two types of interfaces.

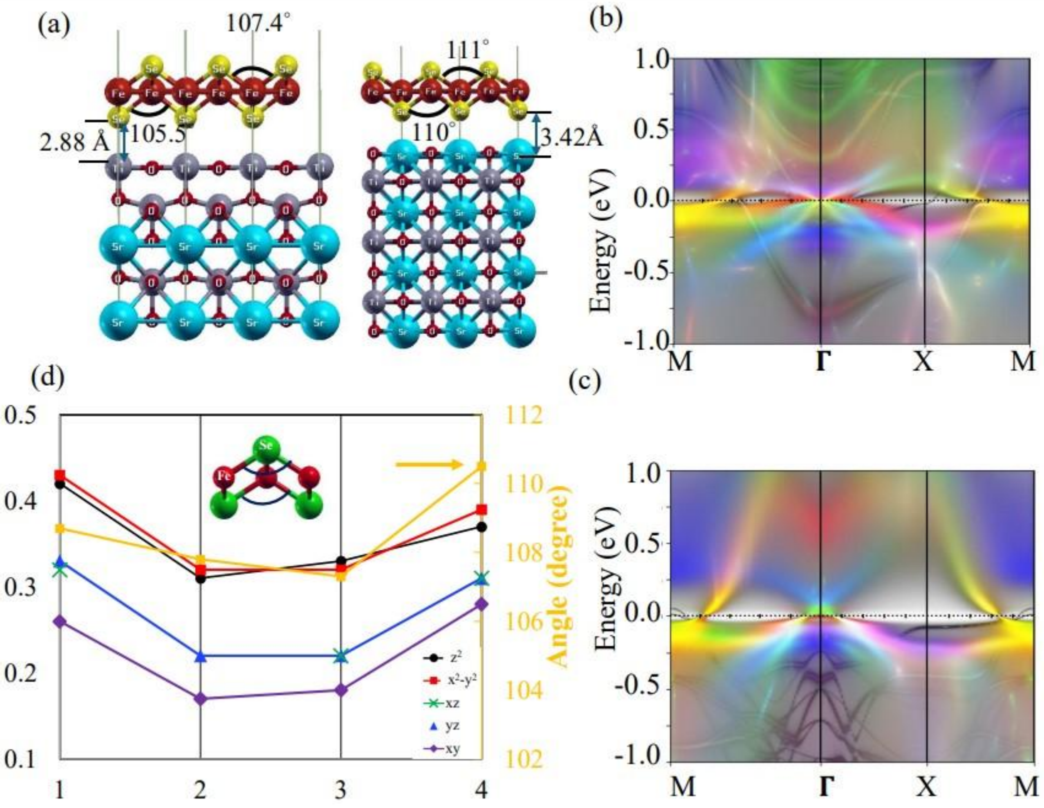

Figure S12 | eDMFT calculated atomic and band structures of single-layer FeSe on TiO_2- and SrO-terminated STO. Ball-and-stick models of the FeSe/STO heterostructures with double TiO_2 and SrO termination. b-c Computed DMFT spectral function for the two heterostructures, respectively. d. Computed spectral weight (Z) on different Fe-3d orbitals (left) and Se-Fe-Se angle (right) show a similar trend across four structures: FeSe on single TiO_2-terminated STO without (1) and with O-vacancy (2), FeSe on double TiO_2-terminated STO with O-vacancy (3), and FeSe on SrO-terminated STO without O-vacancy (4). The inset represents a schematic of the Se-Fe-Se angle in FeSe.
